# Tensile Properties of Four Types of ABS Lattice Structures—A Comparative Study

**DOI:** 10.3390/polym15204090

**Published:** 2023-10-15

**Authors:** Katarina Monkova, Peter Pavol Monka, Romana Hricová, Berenika Hausnerova, Lucia Knapčíková

**Affiliations:** 1 Faculty of Manufacturing Technologies, Technical University in Kosice, 080 01 Presov, Slovakia; peter.pavol.monka@tuke.skromana.hricova@tuke.sk (R.H.); lucia.knapcikova@tuke.sk (L.K.); 2 Faculty of Technology, Tomas Bata University in Zlin, Nam. T.G. Masaryka 275, 760 01 Zlin, Czech Republic; hausnerova@utb.cz

**Keywords:** lattice structure, tensile properties, Acrylonitrile Butadiene Styrene (ABS), additive manufacturing

## Abstract

This article aims to compare the behaviour of four types of lattice structures named Cartesian, Rhomboid, Octagonal, and Starlit under tensile stress loading. The structures were made of Acrylonitrile Butadiene Styrene (ABS) material using the Fused Filament Fabrication (FFF) technique with three different specific volumes (24, 42, and 60%). Five samples of each type were produced, and a total of 60 samples were tested. Experimental testing was performed according to EN ISO 527-1:2012 and EN ISO 527-2:2012. The obtained data were statistically processed, while no outliers were identified. The experimental results pointed out that the specimens’ topology, together with the specific volume, very significantly affected the resultant ABS properties of the tested samples made of the same material. The comparative study showed that in terms of ultimate strength, yield strength, and Young’s modulus, the Cartesian structure appeared to be the most suitable for tensile stress, and the least suitable structure was the Rhomboid structure. On the other hand, the Rhomboid-type of the structure showed not only the highest amount of absorbed energy but also the highest toughness among the investigated lattice structures, so in the near future, its behaviour under an impact test should be studied.

## 1. Introduction

Additive production makes it possible to produce bodies with complex shapes that are difficult to produce using conventional methods of production. This group of so-called metamaterials also includes cellular lattice materials. These structures are commonly found in nature, where they serve to lighten load-bearing structures, for example, in the wings of animals or in plants [1,2]. They have become an inspiration for use in technical practice but the properties of such structures must be known before their application and connection, not only with their topology, but also with the material and production technology, is formed [3,4,5,6]. Thanks to the good mechanical properties of such lightweight structures—including low weight, the good absorption of mechanical energy, and high strength in relation to the mass density—they provide promising potential in various industries (e.g., aerospace and automotive) or other areas such are healthcare or biomedicine, respectively, in social spheres [7].

When applying porous material to the production of components used in technical practice, these, like full-volume material, must meet the pre-required criteria. The resulting mechanical properties and uses of porous structures depend on many factors, with not only the material from which they are made playing an important role but also the topology of included cells and the relative volume ratio of the material in a given structure [8,9,10].

As for the material itself, compared to metals, plastics are more affordable and cheaper, and their mechanical properties, in combination with user needs, are in many cases sufficient for their application not only in household applications but also in stressed components of technical practice [11].

The increasing potential of using light metamaterials and especially the developing possibilities of their production using 3D technologies have aroused worldwide interest in the investigation into the mechanical properties of types of lattice structures [12].

Jimbo and Tateno [13] dealt with the design of isotropic-tensile-strength lattice structures with various sizes made of ABS material. The cubic BCC structure was chosen as the aim structure type because the authors hypothesised that it could reduce anisotropic properties due to the additive manufacturing process. Subsequently, the samples were subjected to tensile tests. However, the result of these tensile tests showed isotropy in tensile strength.

Durbaca et al. [14] experimentally investigated the mechanical behaviour of various polymeric materials, especially ABS (Acrylonitrile–Butadiene–Styrene), PLA (Polylactide), and PC (polycarbonate) with 40% CF (carbon fiber). The samples were made with a fibre thickness of 3 and 5 mm. In particular, the authors investigated a comparative analysis of the effect of increasing the thickness of the core on the strength of the structure. Their experimental tests performed on test samples showed similar mechanical properties, and the effect of increasing the solid composite weight with an increasing core thickness and type of arrangement was found. At the same time, it was proved that the arrangement of the lattice structure influenced the strength of the structure.

Sadali et al. [15], in their research, focused on modelling and manufacturing a 3D lattice structure using 3D printing from a polylactic acid material consisting of three different strut angles, including 45/45, 25/65, and 60/30 degrees. The aim of this study was to determine the tensile behaviour of structures. Subsequently, these structures were experimentally tested according to the ISO 527 standard [16] using a universal testing machine at a displacement speed of 1 mm/min. An analysis of variance (ANOVA) was also performed to prove the accuracy of the result. The 25/65 design was shown to be the best result compared to the other samples tested in this study.

The relative influence of the lattice core and the side parts of the layer (perimeter) on the lattice structures produced by FFF technology was studied analytically and experimentally within the research of An et al. [17]. These two parts were chosen by the authors because they are the main components that resist external forces. The aim of their work was to study the effects of tensile force with respect to the density of the filling, the raster angle, and the thickness of the side layer. They also created an analytical model for a better understanding of measured data. They proved that this model is consistent with the experimental results. These experimental results and predictions showed that the perimeter (side layer) has a much greater effect on the structures than the core of the grid itself, except for a small raster angle. Their further experiments with samples also proved the influence of the fill density, raster angle, and circumferential thickness on mechanical properties.

Farbman and McCoy [18] dealt with the tensile testing of 3D-printed samples made of ABS and PLA material. They experimentally tested 13 samples with a volume ratio of 10, 30, and 50% with different types of structure, orientation, and strain rates. Subsequently, all the results were compared, and it was found that the specific ultimate tensile strength (MPa/g) decreased with the infill percentage of the test sample. However, in the finite element analysis, the hexagonal structure showed lower deformation compared to the case of the cube linear structure. Also, Wu and Yang [19] dealt with a theoretically proposed model for predicting the site of initial failure in lattice structures. The authors investigated the effects of cell size and topology on tensile failure in several representative 2D lattice structures using an analytical model. The results showed that the diamond structure with lower nodal connectivity shows a relatively progressive model of crack propagation compared to the triangular structure.

Xu et al. [20] presented a model of the deformation and fracture behaviour of 3D printed lattice materials under uniaxial tensile loading. The samples were made in two directions of 3D printing (horizontal and vertical). To obtain a pattern of cracks and load curves, uniaxial tensile tests were performed on printed lattice materials. Subsequently, the experimental results and simulations were comparatively analysed. The results emphasise the importance of considering the direction of 3D printing when simulating mechanical performance.

The aim of the work of Raghavendra et al. [21] was to examine three configurations of structures, namely regular (square), irregular (oblique square), and complete random structures on three different porosity levels. Samples were made using the selective laser melting of Ti6Al4V powder. The deviations of the produced samples were evaluated using morphological characteristics and porosity analysis. The samples were subjected to cyclic tensile tests. The results from the study indicate a clear deviation in the thickness values from as-designed values.

Structural defects arising during production that had an influence on determining the tensile strength of hexagonal lattice structures were studied in the research of Seiler et al. [22]. A combination of experiment and finite element analysis was used to interpret the results. It was found that the macroscopic tensile strength of the hexagonal lattice is most sensitive to imperfections in the form of broken cell walls owing to its low transition flaw size. By contrast, only a mild sensitivity of the tensile strength is observed for the cases of misplaced joints and cells filled with solid inclusions.

Hasan et al. [23], in their study, focused on the determination of elastic properties for 3D printed ABS single strut specimens and made a comparison with that of reclaimed carbon fibre. This study was applied for straight struts using the compliance correction method with different gauge lengths of between 8 mm and 30 mm and single fibre. The tensile test was performed according to the standard procedure on the Shimadzu EZ test (EZ-LX) machine. It was found that a reliable modulus was determined for ABS single strut using the compliance correction method; meanwhile, the rCF (refractory Ceramic Fiber) modulus was strongly correlated with the condition of tested fibres.

The use of published values for the elastic modulus of ULTEM-9085 honeycomb lattice structures in an FE simulation with a 40–60% error is shown in the predicted elastic response of the paper by Bhate et al. [24]. A methodology that combines experimental, analytical, and numerical techniques to predict elastic response within a 5% error was developed there. 

The main aim of Kessler et al.’s [25] work was an extraction of important parameters concerning the shape of lattices and different factors that affect lattice structures. In this survey, they focused on mechanical properties and the, therefore, necessary tensile tests of various lattice-structured samples with different struts and cell sizes. Some of the samples were produced with vertical struts. The result highlighted some special abnormalities in samples in relation to the interesting progression of the curve in the tensile test.

Bauer et al. [26] focused on the impact of size and loading direction on the strength of architected lattice materials. In this work, they investigated the strength of polymer alumina core–shell composite micro lattices with different pattern sizes in compression and tension. The results showed that the compressive strength increased by a factor of two when the lattice size was scaled down by 50%. With tensile strengths of up to 27 MPa at 0.37 g/cm^3^, the micro lattices outperformed all technical foams and most monolithic ceramics.

The main aim of the investigation of Kessler et al. [27] was to analyse different parameters that influenced the mechanical properties of lattice structures, focusing on tensile strength. Furthermore, the secondary goal of this research was to find several different parameters that had a positive effect on mechanical properties. Emphasis was placed mainly on the experimental tensile test according to DIN 50125. All results were calculated with the cross-sectional area of the probe and not with the real cross-sectional area of the massive material. The results showed that the massive probes had no conspicuous issues. The lattice probes had the same values (curves overlap). The coated lattice structure indeed had higher tension values in comparison to the lattice probes, but among themselves, they all had a tension of 134 MPa. All curves were linear and showed a continuous transition to the elastic and plastic deformation, whereas the elastic extension area indicated Hook’s law.

As can be seen from the previous summary, despite several existing studies in the area of the tensile stress behaviour of cellular materials, a comparative study on the tensile properties of lattice structures made of Acrylonitrile–Butadiene–Styrene (ABS) material via additive manufacturing including 4 types of topologies with open porous cells (Cartesian, Starlit, Rhomboid, and Octagonal) and 3 relative densities (24, 42 and 60%) has not been realised so far. The main goal of this research was to determine the degree of influence of basic factors (volume ratio and cell typology) on their ultimate tensile strength, yield strength, and Young’s modulus of elasticity. Energy absorption and toughness were also evaluated as important factors in determining the material’s ability to withstand stress and deformation. The obtained results are valuable not only in terms of their implementation in technical practice when they help the designer to decide on the possibility and suitability of their application to lightweight components, but also in terms of numerical analysis, which is a further goal of the authors, as the experimental results form the basis of boundary conditions and for numerical analyses of a similar nature.

## 2. Materials and Methods

### 2.1. Materials

Due to its good availability and excellent mechanical properties, ABSplus-P430 Ivory material, which is one of the most used and best-known thermoplastic polymers in additive manufacturing, was chosen for all samples that were experimentally tested. It is used not only in industrial sectors, where it has wide-spectrum use, but also in the production of various covers and car parts or in research areas in the creation of cheap and accurate prototypes, samples, and demonstration models. It is also used by the general public for the production of various 3D-printed home accessories [28,29].

The development of additive manufacturing enabled the production of complex, shape-complicated, three-dimensional shaped bodies with cavities in the middle. It is, therefore, a promising way of manufacturing lightweight components that contain regular cellular structures. In addition to the material from which these structures are made, a very important factor for cellular structure properties is the so-called specific volume *Sv*, or relative volume fraction, which expresses the percentage of cell space that is filled with material. It is given by the following equation [30]:

(1) $$Sv=\frac{V_{M}}{V_{BC}}\times 100\;\left(\%\right)$$

where *V_M_* is the volume of the material that fills a basic cell and *V_BC_* is the total volume of a basic cell.

### 2.2. Preparation and Production of Samples

For a comparative experimental study on the behaviour of regular cellular structures under tensile loading, 4 types of lattice structures, including Cartesian, Octagonal, Rhomboid, and Starlit, named after the geometry of the basic cells were chosen. The choice of structure type was based on previous research, in which the Cartesian structure with a simple BCC (Body Cantered Cubic) cell dominates most often [31]. Non-traditional and little-used Octagonal, Rhomboid, and Starlit types of structures were compared with this structure. Each type of structure was produced using 3 specific volumes of material: 24, 42, and 60% (with a maximum standard deviation of ±0.5%), which was predominantly controlled by the diameter of the “strut” in the structure. The choice of specific volume was based on the overall dimensions of the designed samples and the capabilities of the 3D printer used; this was available at the authors’ workplace because, for example, samples with a volume fraction of less than 24% could not be produced to a satisfactory quality. The upper limit of 60% was chosen regarding the goal of using these structures, which is to lighten the components while preserving them. To ensure the repeatability and statistical evaluation of the measurements, 5 pieces of samples were tested from each type of structure and from each specific volume. A total of 60 samples were produced (4 types × 3 specific volumes × 5 pieces).

All samples had a cylindrical shape with an outer diameter of 25 mm. The core of the lattice structure of each sample was created by copying the basic cell, while the cross-section was generated in the *xy* plane, and the “*z*” axis was always the same as the axis of the sample’s cylindrical shape (later during production, the *z*-axis was the same as the direction of application for individual layers of the material). Considering the preservation of the specific volumes determined, which is part of the criteria used to compare the behaviour of individual cell types, the diameter of the sample, as well as the capabilities of the testing equipment used, were considered. This included the length of the parallel part of the samples containing the structure, which was set to 90 mm, while, in some samples, it was adjusted to 88 mm due to the requirement of preserving the integrity of all cells located in the sample volume. The ends of the samples determined for their clamping were designed according to the needs of the testing equipment; therefore, the total length of the samples was 200 and 198 mm, respectively. 

More detailed characteristics of the tested samples are presented in Table 1.

The production process of all samples began with the generation of virtual models of these samples with the required dimensions, structures, and volume ratio in the CAD/CAM system environment of the PTC Creo Parametric 8. Subsequently, an STL file was generated in the software, enabling the given models to be printed.

**Figure 1:**
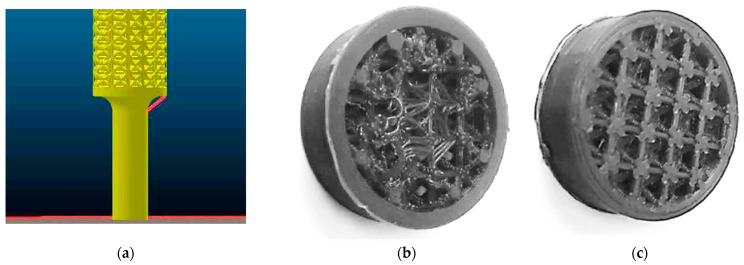
Problems encountered during sample production; (**a**) Problem with radius slicing in Slic3r software Ultimaker Cura 4.7; (**b**) Sample printed with a 0.6 diameter nozzle; (**c**) Sample printed with a nozzle with a diameter of 0.2.

Selected types of lattice structures were produced using the FFF (Fused Filament Fabrication) technique, while ABS material was used in the form of a filament with a diameter of 1.7 mm. A Prusa I3 Mk2 3D printer (Prusa Research, a. s., Prague, Czech Republic) was employed to produce the samples. The samples were printed vertically; that is, the axes of cylindrically shaped samples were perpendicular to the built platform. During sample preparation, a few problems were solved. In the software Slic3r, which was used for preprocessing in the production process of the samples, a problem with the unsuitable design of the samples’ ends occurred. It was necessary to replace the primarily selected round with a conical surface (as seen in Figure 1a) so that the layers could be connected to each other during manufacturing. The quality of the structures was improved by changing technological parameters. The most significant effect on the production of structures was manifested in the diameter of the nozzle. The difference in sample quality when using the 0.6 mm and 0.2 mm nozzle diameters is shown in Figure 1b,c, respectively.

**Figure 2:**
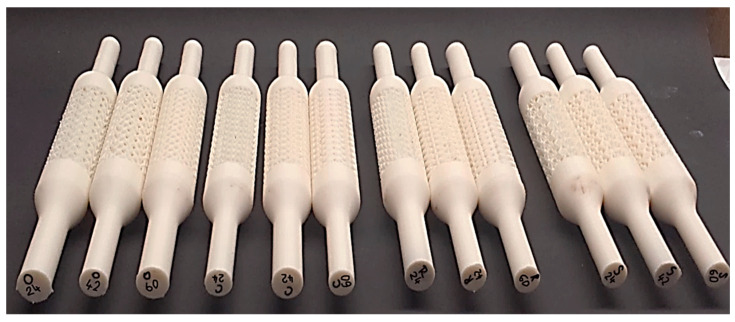
One series of samples with Cartesian (C), Octagonal (O), Starlit (S) and Rhomboid (R) lattice structures with three different specific volumes made of ABS material via FFF technology.

Other parameters were set up as follows. The temperature of the printer nozzle was set to 255 °C, and the built platform was 100 °C. The movement speed of the nozzle with a diameter of 0.2 mm was 40 mm/s (except for the circumference of the sample cross-section, where it was 30 mm/s). The layer thickness determined by the 3D printer manufacturer was set at 0.254 mm. Other parameters were identical to the basic settings of the printer according to the manufacturer’s recommendations. The labelling of the samples was a combination of the type of structure that the samples contained and the specific volume expressed as a percentage, e.g., C24, C42, C60, etc. Figure 2 shows one of five series of produced samples (4 types in 3 specific volumes).

### 2.3. Methods

#### 2.3.1. Testing 

**Figure 3:**
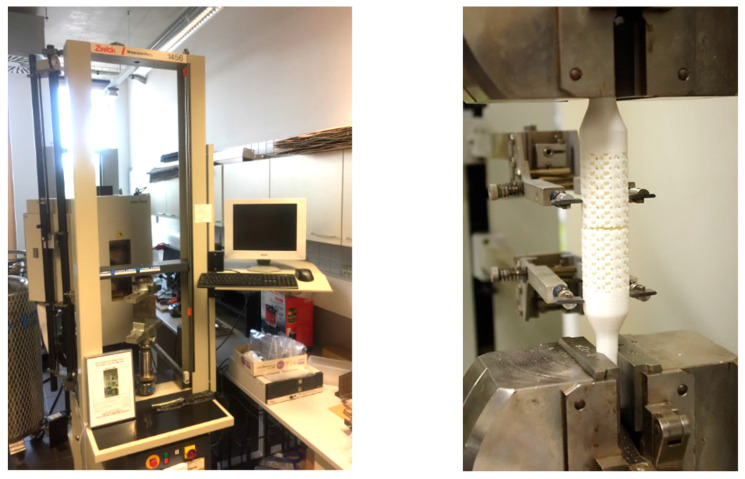
Configuration of the measuring set.

The experiments were carried out on a Zwick 1456 testing machine. This device was used for static and low-cycle dynamic tensile, compressive, bending, and shear experimental tests. The evaluation of the results took place on a connected computer using the TestXpert II software. All tests were carried out according to the standard: EN ISO 527-1:2012 (WI 00249662) [32] and EN ISO 527-2:2012 (WI 00249663) [33] at a laboratory temperature of 22 °C and a relative humidity of 60%. The cross-sectional speed during the measurement of the modulus of elasticity was *v* = 1 mm/min, while during the test itself, it was 20 mm/min. The initial measured length was *l_o_* = 40 mm, and the deformation was measured using a Marco extensometer. The configuration of the measuring set is shown in Figure 3.

The dependences of force (N) on deformation (mm) were plotted during the experimental testing of each structure separately. The smallest cross-sectional area given by the topology of the structure was used to calculate the stress and obtain stress–strain curves. Based on these data, yield strength, ultimate strength, and Young’s modulus of elasticity were determined as the arithmetic mean of five measurements. All obtained results were statistically processed, and a standard deviation was calculated for selected parameters, while the outliers of measured data, determined using the Grubs test of outliers, were not found.

#### 2.3.2. Evaluation 

The evaluation of experimentally obtained data was carried out in several steps and from several points of view.

To obtain the stress–strain curves, the software PTC Creo Parametric 8 was used to precisely specify a cross-section area of the individual type of samples. 

The cross-sectional areas of the structures presented in Table 1 were selected in this way so that a typical arrangement of the structure could be viewed. In real samples, however, the geometry of the cross-section changed within a cell, as did the size of the cross-sectional area. Based on this, the measured data of force load—displacement dependencies were recalculated, and the obtained stress–strain curves were able to identify the ultimate and yield strengths of individual samples as well as Young´s modulus. 

From the point of view of the practical application of cellular structures, an important role is played by the view of how the unit volume of the consumed material is able to bear its load. In this research, the cubic centimetre (cm^3^) was chosen as the basic volume unit, and the average values of the yield and ultimate strength obtained for individual types of samples (characterised by topology and specific volume) were divided by the volume of material consumed.

In general, when selecting materials for a given application, engineers and material scientists consider toughness (J/m^3^) as one of the properties that play an important role in determining this material’s ability to withstand stress and deformation [34]. The formula for toughness depends on the type of loading that the material is subjected to. For tensile loading, the formula for toughness is given by the area under the stress–strain curve. In this case, toughness is calculated as the integral of the stress–strain curve up to the point of fracture [35]:

(2) T=∫(σ dε)

where *T* (J/m^3^) is the toughness, *σ* (Pa) is the stress, and *ε* (-) is the strain.

Therefore, in the next phase, data were processed so that it was possible to evaluate their flexural toughness and energy absorption capacity, including investigating the correlation between them.

The absorbed energy of individual types of samples with a specific volume was calculated as the area under force–displacement plots [36], while the toughness was calculated as the area under the stress–strain curve [37]. This was conducted by integrating the equations obtained for the trend curves in the MS Excel software 2021 application, with the coefficient of determination R^2^ no less than 0.97. 

**Figure 4:**
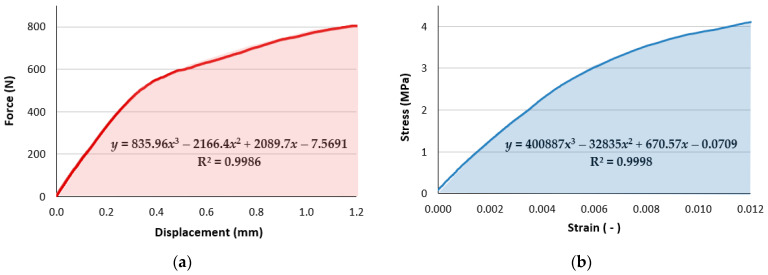
The principle of calculating (**a**) The amount of absorbed energy during the test for the sample R42; (**b**) The toughness of the sample C24.

The principle of calculating the amount of absorbed energy during the test for sample R42 is shown in Figure 4a [38], and the principle of calculation for the toughness of sample C24 is shown in Figure 4b [39].

## 3. Results and Discussion

As part of this comparative study, the results were evaluated from several points of view. Mechanical properties were assessed from three aspects in terms of the influence of the type of structure on the behaviour of the samples under tensile stress, in terms of the influence that the specific volume has on the mechanical properties of individual types of structures, as well as Young’s modulus.

The measured and calculated values of yield strength *Rp*_0.2_, ultimate strength *Rm*, and Young’s modulus of elasticity *E* are shown in Table 2.

### 3.1. Effect of Structure Type

**Figure 5:**
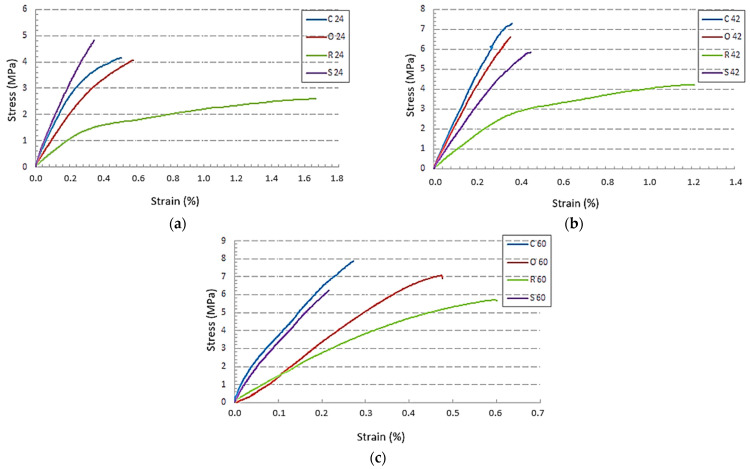
The influence of the type of lattice structure of the material on the behaviouImprovedr of specimens under tensile stress for three different specific volumes *Sv*: (**a**) *Sv* = 24%; (**b**) *Sv* = 42%; (**c**) *Sv* = 60%.

The resultant mechanical properties of porous materials depend primarily on the topology of the structure [40,41]. The effect of structure type on ultimate tensile stresses at three different specific volumes of the material *Sv* is shown in Figure 5.

The results show that from the point of view of ultimate stress, the least suitable structure for tensile stress is the Rhomboid structure. The influence of other types of structures on the behaviour of the samples during tensile stress is not clear from the research, as structural properties alternated with different specific volumes of the material. Due to the fact that the manufacturing and testing conditions of all structures were the same and given that the diameters of the lattice struts (when comparing structures with the same specific volume) were also the same, it is most likely that cell topology plays an important role in the tensile properties of the samples. One of the reasons that could cause the smallest tensile stresses in the Rhomboid structure is the spatial arrangement of struts within the cell, which affects the size of the cross-sectional area of the structure. Since the size of the real cross-area of the structure changes within the cell, the smallest cross-sectional areas are always taken into account for each type of structure in the evaluation. In this case, when the sample is stressed by the same force, the critical points of the structure originate as they generate the greatest stresses. In connection with this consideration, the maximum measured forces were added to Table 2. The recorded maximum forces showed that the forces for the Rhomboid structure were smaller than for the Starlit structure but greater than for the Cartesian and Octagonal structures. In the Rhomboid structure, however, the smallest cross-sectional area was identified for each volume fraction, which was always the largest among the compared structures, and thus, the stresses achieved with the same (or slightly higher) force load were the lowest after recalculation.

From the comparison of these results, it can be concluded that the Cartesian structure appeared to be the most suitable structure for tensile stress, even though in the case of a 24% specific volume, the Starlit structure achieved the best strength limit. The reason for this could be found in the spatial geometry/topology of the cell since technological conditions for the production of all structures investigated in this study were the same. In a deeper consideration of the justification of the highest strength limit for the Starlit 24% structure, it is possible to point to the fact that the crossing and joining of struts create notches that generally have a high effect on the resultant mechanical properties of the components. Notches result in uneven stress distribution, leading to stress peaks—which is known as the notch effect. The notch effect reduces the loading capacity, which makes it a crucial parameter when calculating the mechanical strength of components or constructions. It refers to the phenomenon where a small geometric discontinuity or a notch in a component can significantly increase the local stress, leading to a reduction in the component’s strength and fatigue life [42,43,44]. The presence of a notch can create a localised stress concentration, which can initiate crack propagation and eventually lead to the failure of the component, even under relatively low applied loads [45,46]. The stress concentration in the notches is influenced by many factors, such as the radius of rounding, the angle between struts, etc., which can play a significant role in the resultant mechanical properties of the components. With this Starlit 24% structure, the influence of a combination of mentioned factors was probably the lowest, as reflected in the highest strength limit value. 

**Figure 6:**
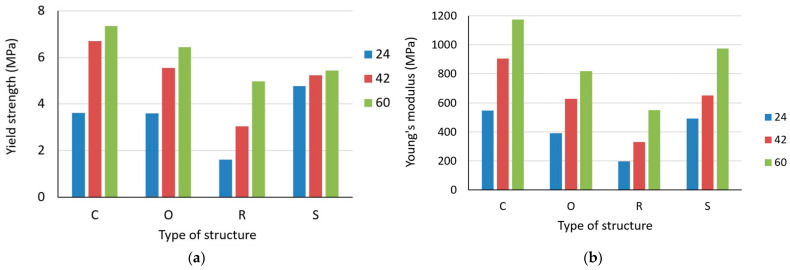
Effect of the type of structure on (**a**) Yield strength; (**b**) Young´s modulus; for all investigated specific volumes *Sv* = 24, 42 and 60%.

An overall view of the impact of the structure type with all three specific volumes *Sv* = 24, 42, and 60% on yield strength and Young´s modulus is presented in Figure 6. From the histograms, it is clear that the Cartesian structure achieved the best overall results for the yield strength and Young’s modulus, whereas the Rhomboid structure performed the worst.

The research hypotheses assumed the definite influence of the specific volume with the same trend for all structures in the sense of improving the measurable indicators of mechanical properties in the structures as the volume fraction increased. Regarding the influence of the cell topology on the mechanical properties in tension, the authors assumed that the order of the structures would be preserved for individual specific volumes (one structure will always show the best properties, another always the worst…). However, research has shown that the resulting mechanical properties are influenced by a combination of both parameters. And since the specific volume was controlled by the diameter of the struts, it confirms what the authors also found with other types of structures (for example, the so-called triply periodic minimal surfaces) [47], that the wall thickness and other properties in relation to stress concentrations at points of connection with individual struts are other factors that are needed to be further investigated and at the same time to verify the already achieved results.

### 3.2. Effect of Specific Volume

**Figure 7:**
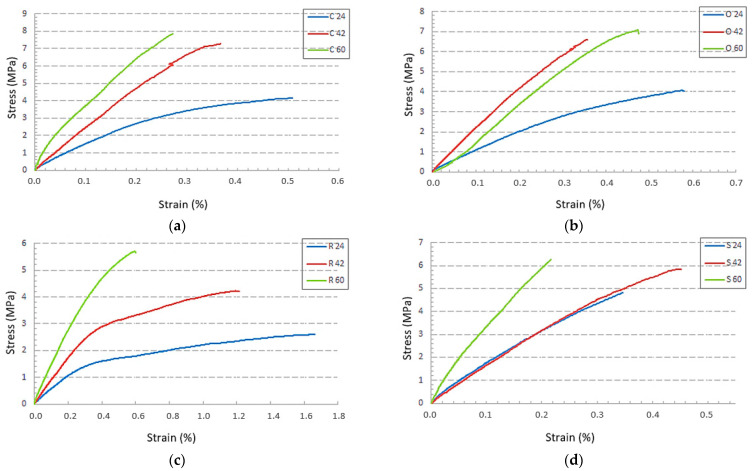
The influence of the specific volume of the material on the behaviour of samples under tensile stress for four types of lattice structures: (**a**) Cartesian; (**b**) Octagonal; (**c**) Rhomboid; (**d**) Starlit.

Another important factor influencing the mechanical properties of structures is the specific volume of the material (Figure 7). In the present research, the volume of material used to produce a sample with a uniformly distributed structure was controlled by increasing the thickness of the cell wall, i.e., the diameter of the struts. In general, the higher the specific volume of the samples with the same structural topology, the higher the mechanical properties [48,49,50]. This assumption was also confirmed in this research, although the differences in inter-strength were very small for samples with specific volumes of 42 and 60%, except for the Rhomboid structure, where the difference in inter-strength between R42 and R60 was the largest. The influence of the specific volume on the strain cannot be clearly described either since the relative elongation decreased with the specific volume in the Cartesian and Rhomboid samples. However, in the Octagonal and Starlit structures with 42% specific volume, relative elongation was the lowest (Figure 7b) or, respectively, the highest (Figure 7d).

When looking at, e.g., Rhomboid structure behaviour (Figure 7c), it can be seen that the samples R24 did not have enough strength and the samples R60 were not tough enough. It can be said that the R42 samples were strong and tough at the same time when we compared them with samples of the same type but with different specific volumes, which was also confirmed by the results presented later in this manuscript. 

The aforementioned theories about the nature of the strong and tough parts at the same time are based on the brittle-to-tough transition related to conventional notched impact strengths [51]. In conventional impact tests, a specimen fracture must occur; otherwise, no values can be determined. This means that only one part of stable crack growth is included in the value for ‘notched impact strength’, and on this basis, it is impossible to separate the stable and unstable parts of the crack propagation process. If this region of predominantly stable crack propagation is reached, the micro-mechanical distortion mechanism follows the assumptions of Margolina described in Dettenmaier [52], i.e., the critical stress for matrix yielding is reduced owing to a change from plane strain to plane stress conditions in the matrix ligaments between particles with decreasing interparticle distance. Through this mechanism, the stress on the struts oriented in the loading direction can be relieved, and the energy dissipation capacity of the material increases. The results of fracture mechanics investigations also indicate the validity of Wu’s percolation theory stated in Lach [53] for the brittle-to-tough transition, according to which the transition from a brittle to tough fracture is connected with a basic change in the distortion mechanism, from crazing to cavity foundation with the local plastic distortion of matrix ligaments. The next transition, from tough to high impact, appears if the whole matrix material is included in the distortion process, i.e., most of the matrix ligaments are oriented in the stress direction and are able to receive the load. In consequence, this process leads to the strong energy dissipation capacity of the material [54].

A solid material undergoes elastic deformation when it is subjected to a small load in compression or extension. Elastic deformation is reversible; that is, the material returns to its original shape after the load is removed. The ability of a material to resist changes in length during longitudinal tension (or compression) is referred to as the modulus of elasticity or Young’s modulus. A higher Young’s modulus corresponds to greater stiffness. Young’s modulus is a fundamental concept in engineering design because it is critical in determining the stiffness of the final product. Its value is, therefore, very important for designers, as well as today, when defining boundary conditions in numerical analyses of the behavior of components under simulated loading and subsequently under stress in real conditions.

**Figure 8:**
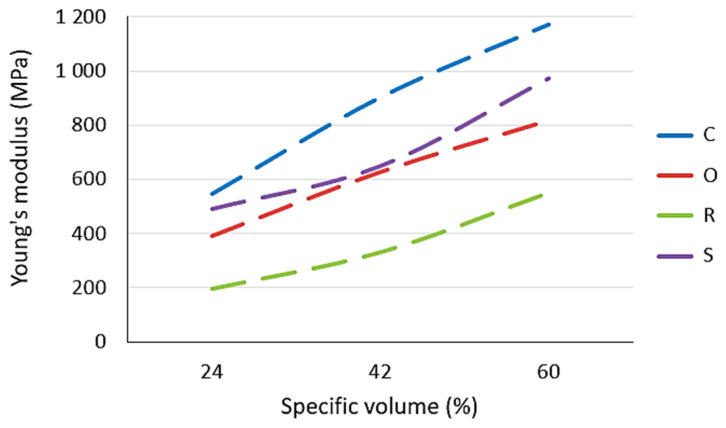
The effect of specific volume on Young´s modulus.

Young’s modulus of the studied lattice structures increases with the increasing specific volume (see Figure 8), while the highest values were achieved by samples with a Cartesian structure and the lowest by samples with a Rhombic structure. 

It can be also said that the curves of dependences of Young’s modulus on the specific volume for the Cartesian, Octagonal, and Rhombic structures show a similar slope of increase, but for the Starlit structure, in the region of 42% of the specific volume, a decrease in values is visible compared to other structures with the same volume fraction.

**Figure 9:**
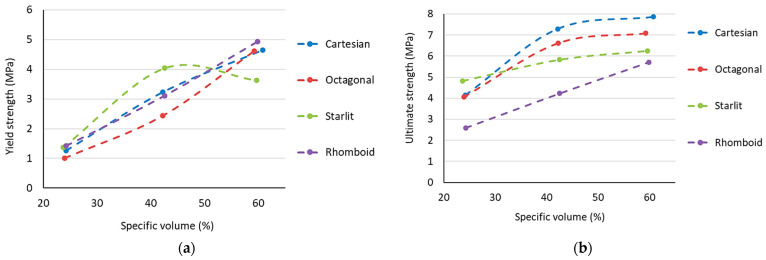
The effect of a specific volume on (**a**) Yield strength; (**b**) Ultimate strength.

To obtain a better understanding of the effect of the specific volume on the strength limit and the yield strength, the achieved values were plotted and are shown in Figure 9. 

From the dependence of the yield strength on the specific volume shown in Figure 9a, it can be seen that the Rhomboid and Cartesian structures had a very similar course, and also, the atypical course of behaviour for the Starlit structure is evident, in which samples with 60% specific volume showed a significant decrease in the yield strength. 

When comparing the ultimate strength limit between these structures, it is clear from the graph in Figure 9b that, in the case of the lowest studied specific volume (24%), the highest strength limit was achieved using the Starlit lattice structure, but the slope of the dependence curve *Rm* for this structure regarding the increasing specific volume was the smallest, which caused the values at higher *Sv* to be lower compared to the Cartesian and Octagonal structures. The strength limit with the highest value in the case of *Sv* = 60% was reached by the Cartesian structure, and the Rhomboid structure showed the lowest *Rm* value in all specific volumes.

**Figure 10:**
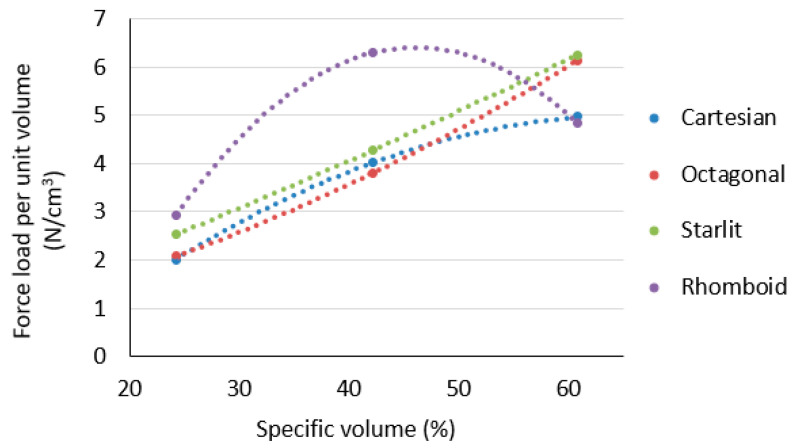
The ability of a unit volume of material to carry a load in tension at investigated specific volumes (*Sv*) of the lattice structures.

The measured and recalculated values of the dependence of stress on the proportional elongation provide an insight into the behaviour of the sample with a multiplied cellular structure under tensile stress, but due to the different specific volumes, they did not provide an insight into how much tensile load the unit volume of this material was able to carry, the data of which are important with respect to the environment and the amount of material consumed. This point of view is expressed by the dependence of the force load per unit volume on the specific volume, as presented in Figure 10. 

It is clear from Figure 10 that the ability of a unit of ABS material at a volume to carry a force load increases as the specific volume of the structure increases. However, this does not apply to the Rhomboid structure. For this structure, the force load per volume unit reached a parabolic trend with a maximum at the specific volume of *Sv* = 42%, but at *Sv* = 60%, the load force per unit volume of the material was the lowest. 

### 3.3. Energy Absorption and Toughness

Since different structures and different specific volumes of these samples are parameters affecting the properties of structures to a relatively large extent, the amount of absorbed energy and toughness were chosen as additional criteria for the mutual comparison of the behaviour of investigated samples under a tensile load.

**Figure 11:**
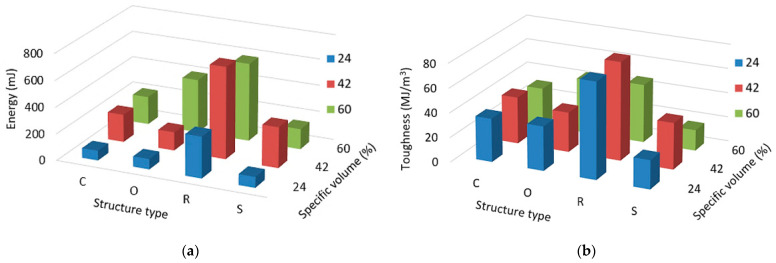
Effect of the structure type and specific volume on (**a**) Energy absorption; (**b**) Toughness; for all investigated specific volumes *Sv* = 24, 42 and 60%.

Tensile energy absorbed is the amount of work that is performed when the material is stretched to rupture under tension. It is a measure of the ability of a material to absorb energy under variable loading conditions [55,56]. Toughness is the strength with which the material opposes rupture and can be expressed as the amount of energy per unit volume (that a material can absorb before rupturing) [57]. A comparison of the amount of energy that was absorbed by individual samples is shown in Figure 11a, and the toughness assessment of the structures is presented in Figure 11b.

Due to the fact that it is a plastic material and also due to the thin struts of these structures, the amount of energy required to fail the samples was small; therefore, it was expressed in millijoules (mJ). 

It would be expected that the samples with the largest specific volume, 60%, would need the most energy for failure [58], but this was not confirmed for the Rhomboid and Starlit structures. Since the measured values in repeated tests were similar to the same type of samples (outliers were not identified during the statistical processing of the data), and the graphs show the average value of five measurements, the behaviour of the samples was confirmed. 

The design or innovation of a new component is always a challenge, and many aspects need to be taken into account. In many operations, it is important that component failures are identified before component failure occurs. In a brittle material, a crack propagates quickly after the defect has formed, which can sometimes cause fatal consequences. The ability of a material to absorb energy and plastically deform without fracturing is toughness, or in other words, it is the resistance of the material to the propagation of a crack. In a hard, brittle material whose toughness is low, while, in strong and ductile material, it is high. From the type of samples point of view, it can be said that the Rhomboid-type structure showed not only the highest amount of absorbed energy but also the highest toughness among the investigated lattice structures. These characteristics are important for many components (e.g., in the automotive industry), and so the implementation of the Rhomboid structure in components with such demanded properties appears as a very reasonable perspective. 

## 4. Conclusions

The essence of the properties of each product is in the material and in the process of its production. These two aspects are interconnected and cannot be separated. However, primary selection usually starts with material based on knowing its behaviour under various conditions. Currently, there is an effort in many industries to produce components that are light and, at the same time, sufficiently tough, meeting the criteria of excellent quality, functionality, and safety. One of these solutions is the implementation of porous structures in component cores, which can bring exceptional properties to the product. It is known that the mechanical properties of different types of structures and their behaviour under specific loads allow the designer to choose not only the right type of structure but also its specific volume of material or the distribution of the structure in the body.

The aim of the research was, therefore, to identify the properties of some types of lattice structures within the framework of a comparative study.

As part of the present research, 60 pieces of cylindrical-type samples of ABS material with four lattice structures (Cartesian, Octagonal, Starlit, and Rhomboid) in three specific volumes (24, 42, and 60%) were produced using FFF technology. The experimental results were statistically processed, while no outliers were identified.

The results of experimental measurements and the obtained values of ultimate strength, yield strength, and Young’s modulus show that the Cartesian structure appears to be the most suitable structure for tensile stress (even though in the case of a 24% specific volume, the Starlit structure achieves the highest ultimate strength), and that the least suitable structure for tensile stress is the Rhomboid structure. 

The ability of a unit of ABS material volume to carry a force load increases as the specific volume of the structure increases. However, this does not apply to the Rhomboid structure. For this structure, the force load per volume unit reached within a parabolic trend is its maximum at the specific volume *Sv* = 42%, but at *Sv* = 60%, the load force per unit volume of the material is the lowest.

Experiments have shown that the ability of a unit volume of ABS material to carry a force load increases with an increase in the specific volume of the structure, which is mainly due to an increase in the thickness of the struts. However, the exception was for the Rhomboid structure, in which the force load per volume unit reached within the parabolic trend a maximum at the specific volume *Sv* = 42%. 

From the type of samples point of view, it can be said that the Rhomboid-type structure showed not only the highest amount of absorbed energy but also the highest toughness among the investigated lattice structures. Since tough materials are able to absorb a large amount of energy before breaking and are often used in applications where impact resistance is important, such as in construction materials or safety equipment, Rhomboid-type specimens appear to be the most suitable for impact-stressed components. This is why they should be investigated by impact tests in the near future.

## Figures and Tables

**Table 1:** Characteristics of 3D-printed samples.

Type of Structure	Specific Volume (%)	Label	Cross-Section View	Diameter of a Strut (mm)	Structure Length (mm)
Cartesian	24	C24	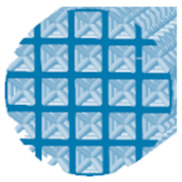	1	90
	42	C42		1.4	90
	60	C60		1.8	90
Octagonal	24	O24	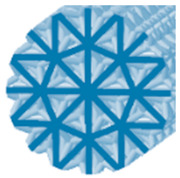	1	90
	42	O42		1.4	90
	60	O60		1.7	88
Rhomboid	24	R24	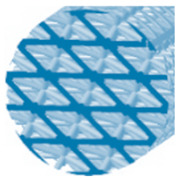	1	88
	42	R42		1.35	90
	60	R60		1.7	90
Starlit	24	S24	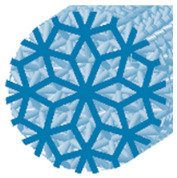	1	88
	42	S42		1.4	90
	60	S60		1.8	88

**Table 2:** Mechanical properties of the investigated 3D-printed lattice structures.

Structure Type	Specific Volume (%)	Label	Force (N)	Yield Strength (MPa)	Ultimate Strength (MPa)	Young’s Modulus (MPa)
Cartesian	24	C24	215 ± 10	3.62 ± 0.22	4.15 ± 0.19	548 ± 12
	42	C42	748 ± 20	6.70 ± 0.24	7.29 ± 0.20	904 ± 22
	60	C60	1193 ± 45	7.35 ± 0.36	7.86 ± 0.30	1173 ± 25
Octagonal	24	O24	220 ± 12	3.60 ± 0.20	4.06 ± 0.17	390 ± 9
	42	O42	709 ± 9	5.55 ± 0.29	6.61 ± 0.33	627 ± 10
	60	O60	1183 ± 56	6.44 ± 0.31	7.08 ± 0.34	817 ± 17
Rhomboid	24	R24	256 ± 9	1.60 ± 0.05	2.60 ± 0.09	197 ± 5
	42	R42	803 ± 23	3.04 ± 0.12	4.23 ± 0.12	331 ± 10
	60	R60	1384 ± 56	4.98 ± 0.27	5.71 ± 0.23	545 ± 16
Starlit	24	S24	306 ± 15	4.77 ± 0.25	4.82 ± 0.24	491 ± 14
	42	S42	934 ± 42	5.23 ± 0.26	5.83 ± 0.26	645 ± 14
	60	S60	1501 ± 70	5.43 ± 0.30	6.25 ± 0.29	974 ± 19

## Data Availability

The data presented in this study are available on request from the corresponding author.
